# Entropy Minimization for Generalized Newtonian Fluid Flow between Converging and Diverging Channels

**DOI:** 10.3390/mi13101755

**Published:** 2022-10-17

**Authors:** Sohail Rehman, Hashim, Abdelaziz Nasr, Sayed M. Eldin, Muhammad Y. Malik

**Affiliations:** 1 School of Material Sciences and Engineering, Georgia Institute of Technology, Atlanta, GA 30318, USA; 2 Department of Mathematics Islamia College, Peshawar 25000, Pakistan; 3 Department of Mathematics and Statistics, University of Haripur, Haripur 22600, Pakistan; 4 Mechanical Engineering Department, College of Engineering and Islamic Architecture, Umm Al-Qura University, P.O. Box 5555, Makkah 21955, Saudi Arabia; 5 Center of Research, Faculty of Engineering, Future University in Egypt, New Cairo 11835, Egypt; 6 Department of Mathematics, College of Sciences, King Khalid University, Abha 61413, Saudi Arabia

**Keywords:** entropy, converging/diverging channel, magnetic field, heat transport, Carreau nanofluid

## Abstract

The foremost focus of this article was to investigate the entropy generation in hydromagnetic flow of generalized Newtonian Carreau nanofluid through a converging and diverging channel. In addition, a heat transport analysis was performed for Carreau nanofluid using the Buongiorno model in the presence of viscous dissipation and Joule heating. The second law of thermodynamics was employed to model the governing flow transport along with entropy generation arising within the system. Entropy optimization analysis is accentuated as its minimization is the best measure to enhance the efficiency of thermal systems. This irreversibility computation and optimization were carried out in the dimensional form to obtain a better picture of the system’s entropy generation. With the help of proper dimensionless transformations, the modeled flow equations were converted into a system of non-linear ordinary differential equations. The numerical solutions were derived using an efficient numerical method, the Runge–Kutta Fehlberg method in conjunction with the shooting technique. The computed results were presented graphically through different profiles of velocity, temperature, concentration, entropy production, and Bejan number. From the acquired results, we perceive that entropy generation is augmented with higher Brinkman and Reynolds numbers. It is significant to mention that the system’s entropy production grew near its two walls, where the irreversibility of heat transfer predominates, in contrast to the channel’s center, where the irreversibility of frictional force predominates. These results serve as a valuable guide for designing and optimizing channels with diverging–converging profiles required in several heat-transfer applications.

## 1. Introduction

The second law of thermodynamics is deemed to be more appropriate from an engineering perspective as compared with the first law. This is because of several factors, including internal friction, vibrating, spin, and molecular kinetic energy allowing heat energy loss that cannot be converted into work. Heat transfer is an energy flow wherein additional motion takes place. Examples of these motions include molecular vibrations, molecule friction, spinning moment, internal movement of molecules, fluid mixing, chemical processes, inelastic distortion of solids, and electric resistivity, etc. Such additional activities result in greater energy loss and entropy. Entropy is a system’s inability to utilize all the available energy efficiently. Entropy generation is a metric for assessing the efficiency of thermal performance and lowering it is important to boost the system’s production. These deficits in the physical world cannot be made up without effort. It is essential to investigate these consequences, which are referred to as irreversibility, inside of any mechanism. Entropy (Ns) is a broad attribute of a thermodynamic process in statistical mechanics. Entropy can only occur in one of three circumstances: case I: (Ns=0) for reversible processes, case II: (Ns<0) for irreversible processes, and case III: (Ns>0) no entropy estimation is possible. For an isolated system, the entropy may be taken as Ns≥0. The second law of thermodynamics is applied, as the amount of accessible work is directly proportional to the amount of entropy generated [1]. As a result, a thermal device that produces less entropy due to irreversibility consumes less energy. This improves the thermal system’s overall efficiency. Consequently, the second law and entropy-generation analysis are frequently employed to assess the causes of irreversibility in diverse components and systems. For instance, in the construction of air-cooled gas turbine engines, the localized rates of entropy production was addressed by Natalini and Sciubba [2]. Kock and Herwig [3] used entropy generation as a tool for evaluating heat-transfer performance in a turbulent shear flow. They created entropy production phrases wall functions and implemented them into a computational fluid dynamic (CFD) code. For an instance, pipe flow with heat flux was investigated and the findings from a direct simulation analysis were compared, with a special focus on entropy generation. The local and global entropy production rates in natural convection in the air in a vertical channel were evaluated numerically by Andreozzi et al. [4]. Later, Makindie [5] investigated the problem of fundamental irreversibility in the flow of a variable-viscosity fluid in a channel with parallel walls and non-uniform temperatures. The inherent irreversibility in a non-uniform (convergent/divergent) channel was examined by Bég and Makindie [6]. After that, Weigand and Birkefeld [7] computed similarity solutions to the Naiver stokes equations with entropy production in Jaffrey–Hamel flow. Furthermore, many investigations considering both Newtonian and non-Newtonian fluid flow through different geometries subject to entropy generation with various physical impacts can be seen in several works [8,9,10,11,12,13,14,15].

The flow of both viscous and non-Newtonian fluids through non-uniform channels having convergent/divergent nature has commanded interest in various fields. Its broad-spectrum applications in industrial, scientific, and manufacturing industries have attracted the interest of several researchers in past few decades. The improvement of the heat-transmission rate in a heat exchanger for milk flow, molten polymer extruded via converging dies, cold drawing operation in the polymer sector, and blood flow through arteries are a few of them. Jeffery [16] and Hamel [17] were the ones who initiated the pioneering work on viscous fluid flow via convergent/divergent channels, a century ago. After that, several researchers addressed this problem under different physical aspects. Hooper et al. [18] analyzed the role of MHD on converging–diverging flow and observed that, in the case of two-dimensional undiluted fluid flow across convergent walls with variable viscosity, velocity interruption increased rapidly as the Reynolds value grows. Makinde and Mhone [19] explored magnetohydrodynamic flows in converging–diverging channels, and it was an extension of Jeffery–Hamel flows to magnetohydrodynamic. He postulated that the external electromagnetic field’s effect serves as a parameter in the solution of MHD flows in convergent–divergent channels. Makinde and Mhone [20] looked in another study that, how tiny disruptions in MHD develop over time and explore the stability of hydromagnetic steady flows in converging–diverging channels at very modest magnetic fields. The critical behavior of the MHD flow in converging–diverging channels was addressed by Alam et al. [21]. Usman et al. [22] evaluated the flow and heat-transfer features of water-based nanofluids within convergent–divergent tubes. Patel and Meher [23] utilized a convergent–divergent channel to analyze the MHD Jeffery–Hamel flow.

Scientists are paying more attention to the evaluation of nanofluids these days. A nano size-particle immersed in the base liquid is a dilute solution with an average size of less than 100 nm, such as water, oils, or ethylene. Such nanoparticles are superior thermal conductors, permitting base fluids to enhance their thermal performance. Choi [24] was the pioneer while introducing nanofluids. Moradi et al. [25] also focused on the consequences of heat transmission and viscosity dissipation on the Jeffery–Hamel flow of nanofluids. Furthermore, Dogonchi and Ganji [26] investigated the impact of velocity and temperature slip on the flow of water-based nanofluids in converging and diverging channels. Extensive reports in recent years have focused on heat-transport mechanisms during nanofluid flow in different scenarios [27,28,29,30,31].

The abovementioned literature suggests that several studies examined the flow of Newtonian and non-Newtonian fluids though converging–diverging channels. However, to the best of the authors’ knowledge, numerical investigation on entropy generation in Jeffery–Hamel flow of Carreau nanofluids has not been presented yet. The authors made a sincere effort in this paper to analyze the impact of the entropy production properties of Carreau nanofluid flowing between two non-parallel walls along with thermophoresis and Brownian motion. It is crucial to note that this kind of surface may be found in a variety of industrial projects where the movement of jet, rocket, and nozzle designs, as well as blood flow in capillaries and arteries, occurs. The flow, energy, concentration, and entropy transport equations for the radial flow of Carreau liquid were formulated under various effects such as magnetic field, viscous dissipation, Joule heating, Brownian diffusion, and thermophoresis diffusion. The leading equations for the flow fields were changed into non-dimensional shapes by using suitable correspondence variables. The important numerical and graphical outcomes were found by solving the non-linear ODEs through the Runge–Kutta Fehlberg technique. Different dimensionless factors, velocity and temperature profiles, and entropy generation and Bejan numbers were numerically explored, and their physical importance was discussed.

## 2. Description and Formulation of the Problem

### 2.1. Physical Configuration

**Figure 1:**
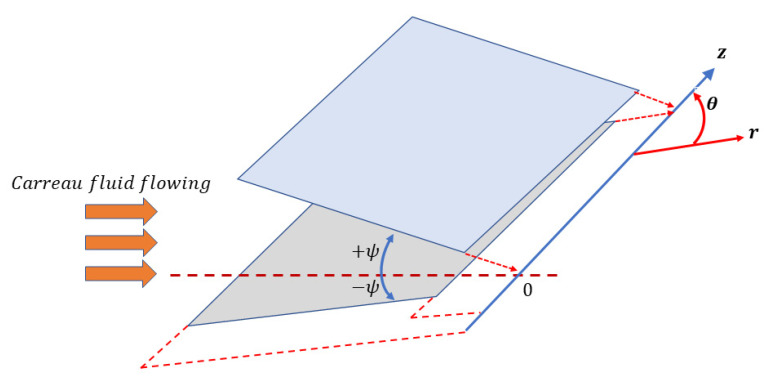
Geometrical configuration of the flow.

A mathematical formulation was modeled for entropy production alongside conservation equations in radial coordinates (r, θ, z). The flow is between two non-parallel flat surfaces having convergent and divergent characteristics, which intersect at an angle 2ψ, as presented in Figure 1. For a purely radial flow, the velocity has only radial component. The flow was taken to be incompressible and is subject to a uniform magnetic field. The uniform magnetic field $\frac{B_{0}}{r}\;$ is acting vertically along the channel walls. Assume that the domain of the investigated flow is −|ψ|<θ<|ψ|, Therefore, the channel’s semi-angle will be ψ. 

The governing transport equations under the above-stated assumptions in their vector form are expressed as:

(1) $$\nabla \cdot \vec{q}=0,$$

(2) $$\left(\vec{q}\cdot \nabla \right)\cdot \vec{q}=div\;\vec{\tau }+J\times B,$$

(3) $$\left(\vec{q}\cdot \nabla \right)T=\frac{k_{f}}{{\left(\rho c\right)}_{f}}\nabla^{2}T+\frac{{\left(\rho c\right)}_{p}}{{\left(\rho c\right)}_{f}}\left[D_{B}\left(\nabla C\cdot \nabla T\right)+D_{T}\frac{\nabla T\cdot \nabla T}{T_{w}}\right]+\Phi +\frac{J\cdot J}{\sigma_{f}},$$

(4) $$\left(\vec{q}\cdot \nabla \right)C=D_{B}\nabla^{2}C+\frac{D_{T}}{T_{w}}\nabla^{2}T.$$

where $\vec{q}=u\left(r,\theta \right)\hat{i}$ denotes the velocity field, $\rho_{f}$ is the fluid density, $k_{f}$, μ, ν, σ, $D_{B}$,$\;D_{T}$, Φ,$\;T_{w}$, and $C_{w}\;$ describs the thermal conductivity, fluid dynamic viscosity, kinematic viscosity, electrical conductivity, Brownian diffusion coefficient, thermophoretic diffusion coefficient, dissipative term, wall temperature, wall concentration, and the ratio of heat capacity of the nanoparticle to the fluid respectively.

The constitutive relation for the non-Newtonian Carreau model is given as follows [32]:

(5) $$T=-pI+\mu A_{1},$$

where

(6) $$\mu =\mu_{f}{\left[1+{\left(\Gamma \dot{\gamma }\right)}^{2}\right]}^{\frac{n-1}{2}}.$$

Here n, indicates the power–index ranges from 0<n<1, refers to pseudoplastic or shear-thinning fluids, while n>1, displays the dilatant or shear thickening fluids, Γ represents the material parameter, and $A_{1}$ denotes first Rivlin–Erickson tensor.

The strain rate under the assumed flow field takes the following form:

(7) $$\dot{\gamma }=\sqrt{2{\left(u_{r}\right)}^{2}+\frac{1}{r^{2}}{\left(u_{\theta }\right)}^{2}+\frac{2u^{2}}{r^{2}}}.$$

While the viscous dissipation term can take the following form:

(8) $$\Phi =\mu_{f}{\left[1+\Gamma^{2}\left\{2{\left(u_{r}\right)}^{2}+\frac{1}{r^{2}}{\left(u_{\theta }\right)}^{2}+\frac{2u^{2}}{r^{2}}\right\}\right]}^{\frac{n-1}{2}}\left(2{\left(u_{r}\right)}^{2}+\frac{1}{r^{2}}{\left(u_{\theta }\right)}^{2}+\frac{2u^{2}}{r^{2}}\right).$$

Based upon these facts, the flow equations in view of the basic conservation laws reduces to

Mass conservation:

(9) $$\rho_{f}\left(u_{r}+\frac{u}{r}\right)=0,$$

Momentum conservation:

(10) $$\begin{array}{cc} \rho_{f}\left(uu_{r}\right)=-p_{r} & +\mu_{f}[\frac{1}{r}\frac{\partial }{\partial r}\left\{r{\left(1+\Gamma^{2}\{2{\left(u_{r}\right)}^{2}+\frac{1}{r^{2}}{\left(u_{\theta }\right)}^{2}+\frac{2u^{2}}{r^{2}}\right)}^{\frac{n-1}{2}}2u_{r}\right\}\\ & +\frac{1}{r}\frac{\partial }{\partial \theta }\left\{{\left(1+\Gamma^{2}\{2{\left(u_{r}\right)}^{2}+\frac{1}{r^{2}}{\left(u_{\theta }\right)}^{2}+\frac{2u^{2}}{r^{2}}\right)}^{\frac{n-1}{2}}\frac{1}{r}u_{\theta }\right\}\\ & +\frac{1}{r}\left({\left(1+\Gamma^{2}\{2{\left(u_{r}\right)}^{2}+\frac{1}{r^{2}}{\left(u_{\theta }\right)}^{2}+\frac{2u^{2}}{r^{2}}\right)}^{\frac{n-1}{2}}\right)\left(u_{\theta }-2u\right)], \end{array}$$

$$0=-\frac{1}{\rho_{f}r}p_{\theta }+\nu_{f}\left[\begin{array}{c} \frac{1}{r^{2}}\frac{\partial }{\partial r}\left\{r^{2}{\left(1+\Gamma^{2}\{2{\left(u_{r}\right)}^{2}+\frac{1}{r^{2}}{\left(u_{\theta }\right)}^{2}+\frac{2u^{2}}{r^{2}}\right)}^{\frac{n-1}{2}}\frac{1}{r}u_{\theta }\right\}\\ +\frac{1}{r}\frac{\partial }{\partial \theta }{\left(1+\Gamma^{2}\{2{\left(u_{r}\right)}^{2}+\frac{1}{r^{2}}{\left(u_{\theta }\right)}^{2}+\frac{2u^{2}}{r^{2}}\right)}^{\frac{n-1}{2}}\frac{2u}{r} \end{array}\right].$$

The continuity Equation (9) suggests that the velocity is purely radial, which depends on r and θ. On simple integration of Equation (9), from −|ψ|<θ<|ψ|, the radial velocity originates as

(11) $$u\left(r,\theta \right)=\frac{H\left(\theta \right)}{r}.$$

Incorporating Equation (12) into the above-shown equations and eliminating the pressure term, the momentum equations reduce to

(12) $$\begin{array}{c} \left(H^{\prime \prime \prime }+4H^{\prime }\right)\;{\left[1+\frac{\Gamma^{2}}{r^{4}}\left({H^{\prime }}^{2}+4H^{2}\right)\right]}^{\frac{{}^{n-1}}{2}}+\frac{2HH^{\prime }}{\nu_{f}}-\frac{\sigma B_{o}^{2}H^{\prime }}{\rho_{f}\nu_{f}}+\frac{\left(n-1\right)\Gamma^{2}}{r^{4}}{\left[1+\frac{\Gamma^{2}}{r^{4}}\left({H^{\prime }}^{2}+4H^{2}\right)\right]}^{\frac{{}^{n-3}}{2}}\times \\ \left(3H^{\prime }{H^{\prime \prime }}^{2}+32HH^{\prime }H^{\prime \prime }+{H^{\prime }}^{2}H^{\prime \prime \prime }+64H^{\prime }H^{2}\right)+\frac{\left(n-1\right)\;\left(n-3\right)\Gamma^{4}}{r^{8}}{\left[1+\frac{\Gamma^{2}}{r^{4}}\left({H^{\prime }}^{2}+4H^{2}\right)\right]}^{\frac{{}^{n-5}}{2}}\times \\ \left({H^{\prime }}^{3}{H^{\prime \prime }}^{2}+16H{H^{\prime }}^{3}H^{\prime \prime }+32H^{3}H^{\prime }H^{\prime \prime }+16H^{2}{H^{\prime }}^{3}+64H^{4}H^{\prime }-4{H^{\prime }}^{5}\right)=0.\; \end{array}$$

The energy and concentration Equations (3) and (4) become

Energy equation:

(13) $$uT_{r}=\frac{k}{{\left(\rho c_{p}\right)}_{f}}\left[\frac{1}{r}T_{r}+T_{rr}+\frac{1}{r^{2}}T_{\theta \theta }\right]+\tau \left[D_{B}\left[T_{r}C_{r}+\frac{1}{r^{2}}T_{\theta }C_{\theta }\right]+\frac{D_{T}}{T_{\infty }}\left[{\left(T_{r}\right)}^{2}+\frac{1}{r^{2}}{\left(T_{\theta }\right)}^{2}\right]\right]\;+\frac{\mu_{f}}{{\left(\rho c_{p}\right)}_{f}}{\left[1+\Gamma^{2}\left\{2{\left(u_{r}\right)}^{2}+\frac{1}{r^{2}}{\left(u_{\theta }\right)}^{2}+\frac{2u^{2}}{r^{2}}\right\}\right]}^{\frac{n-1}{2}}\left[\left\{2{\left(u_{r}\right)}^{2}+\frac{1}{r^{2}}{\left(u_{\theta }\right)}^{2}+\frac{2u^{2}}{r^{2}}\right\}\right]+\frac{\sigma B_{0}{}^{2}u^{2}}{{\left(\rho c\right)}_{f}r^{2}}.$$

Concentration equation:

(14) $$uC_{r}=D_{B}\left(\frac{1}{r}C_{r}+C_{rr}+\frac{1}{r^{2}}C_{\theta \theta }\right)+\frac{D_{T}}{T_{\infty }}\left(\frac{1}{r}T_{r}+T_{rr}+\frac{1}{r^{2}}T_{\theta \theta }\right),$$

With related constraints at the boundaries

(15) $$\left\{\begin{array}{c} u=U,\;u_{\theta }=C_{\theta }=T_{\theta }=0,\;at\;\theta =0\\ u=0,\;T=T_{w},C=C_{w},\;at\;\theta =\psi \end{array}\right\},$$

### 2.2. Similarity Solutions

The similarity solutions are established via well-known dimensionless variables [33]

(16) $$f\left(\eta \right)=\frac{H\left(\theta \right)}{rU},\;\eta =\frac{\theta }{\psi }\;,\;\beta \left(\eta \right)=\frac{T}{T_{w}},\;\gamma \left(\eta \right)=\frac{C}{C_{w}}\;,$$

With the aid of these similarity transformations, Equations (13)–(15) reduce to

(17) (fηηη+4ψ2fη) [1+We2(4ψ2f2+fη2)]n−12+2ψReffη−ψ2M2fη+(n−1) We2×[1+We2(4ψ2f2+fη2)]n−32(3fηfηη2+32ψ2ffηfηη+fη2fηηη+64ψ2fηf2)+(n−1) (n−3) We4×[1+We2(4ψ2f2+fη2)]n−52(fηη2fη3+16ψ2ffη3fηη+32ψ4f3fηfηη+16ψ4f2fη3+64ψ6f4fη−4ψ2fη5)=0, 

(18) $$\beta_{\eta \eta }+\mathrm{Pr}(N_{B}\beta_{\eta }\gamma_{\eta }+N_{T}\beta_{\eta }{}^{2})+PrEc\left[{\left(1+We^{2}\left(4\psi^{2}f^{2}+f_{\eta }{}^{2}\right)\right)}^{\frac{n-1}{2}}\right]\left(4\psi^{2}f^{2}+f_{\eta }{}^{2}\right)+\psi^{2}M^{2}PrEcf^{2}=0,$$

(19) $$\gamma_{\eta \eta }+\frac{N_{T}}{N_{B}}\beta_{\eta \eta }=0.$$

In conjunction with dimensionless boundary conditions

(20) $$\begin{array}{c} f\left(0\right)=1,\;f_{\eta }\left(0\right)=0,\;f\left(1\right)=0,\;\\ \beta \;\left(1\right)=1,\;\beta_{\eta }\left(0\right)=0,\\ \gamma \;\left(1\right)=1,\;\gamma_{\eta }\left(0\right)=0. \end{array}\}.$$

The leading flow parameters are as follows:

$Re=\frac{\psi rU}{v}$, $We^{2}=\frac{\Gamma^{2}U^{2}}{r^{2}\psi^{2}}$, $M^{2}=\frac{\sigma B_{0}{}^{2}}{\rho_{f}\upsilon_{f}}$, $Pr=\frac{\mu \rho_{f}C_{p}}{k_{f}}$, $Ec=\frac{U^{2}}{T_{w\;}c_{p}}$, $N_{B}=\frac{\tau D_{B}C_{w}}{\nu }$, and $Nt=\frac{\tau D_{T}T_{w}}{\upsilon T_{\infty }}$ demonstrate the Reynolds, Weissenberg, Magnetic, Prandtl, and Eckert numbers and the Brownian diffusion thermophoretic parameter, respectively. Additionally, n indicates the shear-thinning and shear-thickening behavior of the Carreau model.

### 2.3. Entropy Generation within the System

Entropy generation implies wastage; therefore, controlling entropy accumulation is frequently a primary goal in modern engineering. Entropy generation assessment can be used to identify the causes of wastage in any system and can be used to optimize the effectiveness of any physical device. The scarcity of universal energy supplies necessitates a reassessment of energy consumption and production practices. The second law of thermodynamics was employed to evaluate energy-generating, -exchanging, and -utilizing systems from a scientific perspective. A nanoparticle’s volumetric rate of local entropy generation is written in terms of thermal transport, viscous dissipation, diffusive irreversibility, and a magnetic field. In vector notation, the entropy rate can be expressed as follows [1,14]:

(21) $$S_{gen}=\underset{\mathrm{Heat}-\mathrm{transfer}\;\mathrm{irreversibility}\;}{\underbrace{\frac{\kappa_{f}}{T_{w}{}^{2}}{\left(\nabla T\right)}^{2}}}+\underset{\begin{array}{c} \mathrm{Viscous}\;\mathrm{dissipition}\\ \mathrm{irreversibility} \end{array}}{\underbrace{\frac{\mu_{f}}{T_{w}}\Phi }}+\underset{\mathrm{Diffusive}\;\mathrm{irreversibility}}{\underbrace{\frac{D_{B}}{C_{w}}{\left(\nabla C\right)}^{2}+\frac{D_{B}}{T_{w}}\left(\nabla C.\nabla T\right)}}+\underset{\begin{array}{c} \mathrm{Joul}\;\mathrm{heating}\\ \mathrm{irreversibility} \end{array}}{\underbrace{\frac{J.J}{\sigma T_{w}}}}.$$

In polar coordinates, the above equation can be put forward as

(22) $$S_{gen}=\frac{\kappa_{f}}{T_{w}{}^{2}}\left[{\left(T_{r}\right)}^{2}+\frac{1}{r^{2}}{\left(T_{\theta }\right)}^{2}\right]+\frac{\mu_{f}}{T_{w}}\left[\left\{2{\left(u_{r}\right)}^{2}+\frac{1}{r^{2}}{\left(u_{\theta }\right)}^{2}+\frac{2u^{2}}{r^{2}}\right\}\right]{\left[1+\Gamma^{2}\left\{2{\left(u_{r}\right)}^{2}+\frac{1}{r^{2}}{\left(u_{\theta }\right)}^{2}+\frac{2u^{2}}{r^{2}}\right\}\right]}^{\frac{n-1}{2}}\;+\frac{D_{B}}{C_{w}}\left[{\left(C_{r}\right)}^{2}+\frac{1}{r^{2}}{\left(C_{\theta }\right)}^{2}\right]+\frac{D_{B}}{T_{w}}\left[T_{r}C_{r}+\frac{1}{r^{2}}T_{\theta }C_{\theta }\right]+\frac{\sigma B_{0}{}^{2}u^{2}}{T_{w}},$$

The dimensionless entropy generation rate with the procedure of similarity variables reduces to

(23) $$Ns=\frac{r^{2}\psi^{2}S_{gen}}{\kappa_{f}}=\beta_{\eta }{}^{2}+Br\left[{\left(1+We^{2}\left(4\psi^{2}f^{2}+f_{\eta }{}^{2}\right)\right)}^{\frac{n-1}{2}}\right]\left(4\psi^{2}f^{2}+f_{\eta }{}^{2}\right)+Md\left(\gamma_{\eta }{}^{2}+\beta_{\eta }\gamma_{\eta }\right)+\psi^{2}BrM^{2}f^{2},$$

(24) $$\mathrm{Ns}=N_{T}+N_{V}+N_{D}+N_{M},$$

where

(25) $$\{\begin{array}{c} N_{T}=\beta_{\eta }{}^{2},\\ \;N_{V}=Br\left[{\left(1+We^{2}\left(4\psi^{2}f^{2}+f_{\eta }{}^{2}\right)\right)}^{\frac{n-1}{2}}\right]\left(4\psi^{2}f^{2}+f_{\eta }{}^{2}\right),\\ N_{D}=Md\left(\gamma_{\eta }{}^{2}+\beta_{\eta }\gamma_{\eta }\right),\;\\ \;\;N_{M}=\psi^{2}BrM^{2}f^{2}, \end{array}\}.$$

Here Br and Md denote the Brinkman number and constant parameter.

(26) $$Br=PrEc,\;Md=\frac{D_{B}C_{w}}{\kappa_{f}}.$$

### 2.4. Irreversibility Distribution Ratio

Bejan [1] established the irreversibility distribution ratio as $\Delta =N_{V}+N_{D}+N_{M}/N_{T}$ to determine whether fluid friction exceeds heat-transfer irreversibility or conversely. When 0≤Δ<1, heat transmission uplifts, and when Δ>1, fluid friction rises. The ratio of entropy generation due to the heat exchange with the entropy generation number is known as the Bejan number Be, which is calculated as follows:

(27) $$Be=\frac{N_{T}}{Ns}=\frac{N_{T}}{N_{T}+N_{V}+N_{D}+N_{M}}=\frac{1}{1+\Delta }.$$

In fact, the Bejan number varying from 0 to 1 is significant. When Be=0, the influence of fluid friction is dominant over irreversibility. At Be=1, the flow system is dominated by irreversibility because of heat transfer. When Be=0.5, the inputs of heat transmission and fluid friction to the generation of entropy are equal.

### 2.5. Curiosity in Physical Measurements

In this study, skin friction coefficient $\;C_{f}$, local Nusselt number Nu, and local Sherwood number Sh were the quantities of engineering importance, which are mathematically written as:

(28) $$\;C_{f}=\frac{\tau_{w}}{\rho_{f}U^{2}}\;,$$

(29) $$Nu=\frac{rq_{w}}{k_{f}T_{w}},$$

(30) $$Sh=\frac{rj_{w}}{DC_{w}}.$$

Here, the wall shear stress $\tau_{w},$ heat $q_{w},\;$ and mass flux $j_{w}$ at the wall are given by

(31) $$\tau_{w}={\frac{\mu_{f}}{r}{\left[1+\Gamma^{2}\left\{2{\left(\frac{\partial u_{r}}{\partial r}\right)}^{2}+\frac{1}{r^{2}}{\left(\frac{\partial u_{r}}{\partial \theta }\right)}^{2}+\frac{2u_{r}{}^{2}}{r^{2}}\right\}\right]}^{\frac{n-1}{2}}\frac{\partial u_{r}}{\partial \theta }|}_{\theta =\psi \;},$$

(32) $$q_{w}={-k_{f}\left(\frac{\partial T}{\partial \theta }\right)|}_{\theta =\psi },$$

(33) $$j_{w}={-D\left(\frac{\partial C}{\partial \theta }\right)|}_{\theta =\psi }.$$

Applying the transformations (13) and (14), the dimensionless form of these quantities become

(34) $$\;C_{f}=\frac{1}{Re}\left[{\left(1+We^{2}\left(4\psi^{2}f^{2}+f_{\eta }{}^{2}\right)\right)}^{\frac{n-1}{2}}f_{\eta }\right],$$

(35) $$Nu=-\frac{1}{\psi }\beta_{\eta }\left(1\right),$$

(36) $$Sh=-\frac{1}{\psi }\gamma_{\eta }\left(1\right).$$

## 3. Numerical Scheme for the Solution

Utilizing the Runge–Kutta Fehlberg method via the MATLAB program, the determining Equations (18)–(20) in conjunction with the boundary conditions Equation (21) were numerically solved. The linked equations and the absence of boundary conditions are the two key justifications for utilizing this method. To guess the missing conditions, we first turned our system of differential equations into a first-order initial value problem. Let us consider

(37) $$f=x_{1},\;f_{\eta }=x_{2},\;f_{\eta \eta }=x_{3},$$

(38) $$\mathrm{Then},\;\mathrm{Equation}\;(18)\;\mathrm{becomes}\;x_{3}{}^{\prime }=\frac{c_{5}}{c_{4}}-\frac{c_{6}}{c_{4}}-\frac{c_{7}}{c_{4}}-\frac{c_{8}}{c_{4}}+\frac{c_{9}}{c_{4}}.$$

where

(39) $$\begin{array}{c} c_{1}={\left[1+We^{2}\left(x_{2}{}^{2}+4\psi^{2}x_{1}{}^{2}\right)\right]}^{\frac{\left(n-1\right)}{2}}\\ c_{2}={\left[1+We^{2}\left(x_{2}{}^{2}+4\psi^{2}x_{1}{}^{2}\right)\right]}^{\frac{\left(n-3\right)}{2}}\\ c_{3}={\left[1+We^{2}\left(x_{2}{}^{2}+4\psi^{2}x_{1}{}^{2}\right)\right]}^{\frac{\left(n-5\right)}{2}}\\ c_{4}=\left[c_{1}+\left(n-1\right)We^{2}c_{2}x_{2}{}^{2}\right] \end{array}\}$$

(40) {c5=−4ψ2x2c1c6=2ψRex1x2c7=(n−1) We2c2[3x1x32+32ψ2x1x2x3+64ψ4x2x12]c8=[x23x32+16ψ2x1x23x3+32ψ4x13x2x3+16ψ4x12x23+64ψ6x12x2−4ψ2x25]c9=−ψ2M2x2} ,

(41) $$x_{4}=\beta ,\;x_{5}=\beta_{\eta },\;x_{6}=\gamma ,\;x_{7}=\gamma_{\eta }$$

(42) $${x^{\prime }}_{5}=-Pr\left(Nbx_{5}x_{7}+Ntx_{5}{}^{2}\right)-PrEc\chi_{1}\left(4\psi^{2}x_{1}{}^{2}+x_{2}{}^{2}\right)-\psi^{2}M^{2}PrEcx_{1}{}^{2}$$

(43) $$x_{7}{}^{\prime }=-\frac{Nt}{Nb}x_{5}$$

with reduced boundary conditions

(44) $$\begin{array}{c} x_{1}\left(0\right)=1,x_{2}\left(0\right)=0,x_{1}\left(1\right)=0,\\ x_{4}\left(1\right)=1,\;x_{5}\left(0\right)=0.\\ x_{6}\left(1\right)=1,\;x_{7}\left(0\right)=0 \end{array}\}$$

The Runge–Kutta Fehlberg integration scheme was used in conjunction with the initial guess values for $f_{\eta \eta }\left(0\right),$ $\beta_{\eta }\left(0\right)$, and $\gamma_{\eta }\left(0\right)$ to arrive at the solution. Then, using the shooting iteration strategy, we altered the values of $f_{\eta \eta }\left(0\right),$ $\beta_{\eta }\left(0\right)$, and $\gamma_{\eta }\left(0\right)$ to provide a better approximation for the solution by comparing the computed values of f(0), β(0), and γ(0) at η=1, with the supplied boundary conditions f(1), β(1), and γ(1). The procedure was repeated until the results were reliable to the required level of $10^{-8}$ accuracies, satisfying the convergence requirement.

## 4. Results and Discussion

### 4.1. Consequences of the Reynolds Number

**Figure 2:**
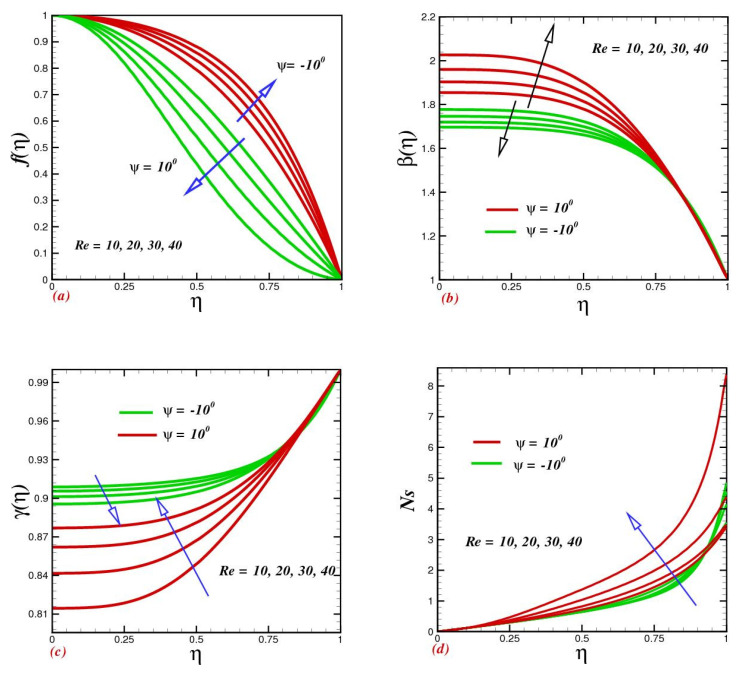
Impacts of the Reynolds number Re on (**a**) velocity f(η), (**b**) temperature β(η), (**c**) concentration γ(η), and (**d**) entropy production Ns.

The growing Reynolds numbers Re on velocity f(η), temperature β(η), concentration γ(η), and entropy production Ns, within the system, are depicted in (a), (b), (c), and (d). Figure 2a reveals that flow velocity climbed as the Reynolds number increased in convergence channels. While for expanding/divergent channels the prescription is converse. Physically, small Reynolds numbers mean that viscous forces are prominent, which means that the flow will be retarded by the development and extension of the boundary layer into this regime. A low Reynolds number means that the viscous force predominates, which signifies that the flow will decelerate since the boundary layer that forms does not reach far into the flow region. Thus, high Reynolds numbers are indicative of turbulent flow patterns, such as those seen in turbulent flows. The Reynolds number elevates heat transfer, as shown in Figure 2b. Temperature configurations for converging/extending and narrowing channels are contrasting. The dropping of temperature in the convergence case was witnessed from the green curves. This is because, as the Reynolds number rises, the viscous force becomes less significant, resulting in reduced fluid viscosity. Due to their inverse relationship, decreasing viscosity inevitably leads to an increase in temperature and vice versa. Thus, the heat progression in narrowing channels is clear. Figure 2c depicts a variety of concentration sketches for a variety of physical parameters. Diverse values of Re depict diverse sketches for concentration in the converging and diverging channels. The concentration of nanoparticles in the divergent channel is stimulated by elevating Reynold numbers. Physically, escalating Re values create inertia, which drives the concentration field to expand in a divergent orientation. Entropy generation rates increase quickly along the two walls with rising Re values as shown in Figure 2d for the oblique channel, which is consistent with the flow-reversal results that are observed in that location. With increasing Re, the rate of entropy formation increased in the vicinity of narrowing and diverging regions. The entropy generation was at its lowest along the centerline of the channel in a particular flow thickness range, relying on the Re. According to this study, there was a minimal entropy generation zone along the channel wall on both sides of the channel.

### 4.2. Consequences of the Weissenberg Number

**Figure 3:**
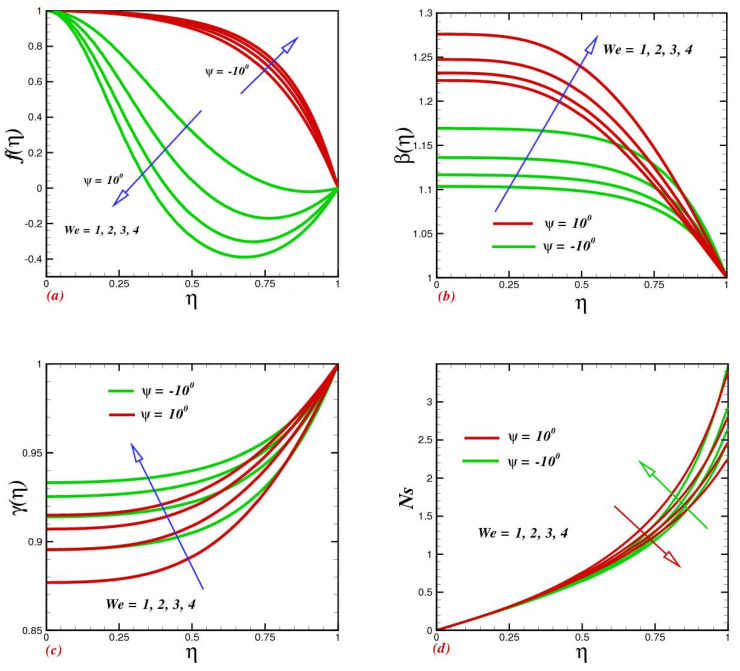
Impacts of the Weissenberg number We on (**a**) velocity *f*(*η*), (**b**) temperature *β*(*η*), (**c**) concentration *γ*(*η*), and (**d**) entropy production Ns.

Figure 3 displays the flow, heat transfer, mass concentration, and entropy generation for dominant values of Weissenberg numbers We in narrowing and extending channels. Fluid flow within diverse geometries seemed diverse against escalating We. Velocity curves uplifted when improving within the range 1≤We<5 for the converging channel however, in another portion, a drastic decline was observed. Growing We upsurges the time constant-to-viscosity ratio, enhancing the Carreau fluid velocity and uplifting the heat of the fluid within the channels, as shown in Figure 3b. Nanoparticle concentration upsurges with escalating We. This justification is due to the lagging values of improving the momentum and thermal boundary layer thickness; as a result, the concentration improves. The heat loss was especially dominant in the converging channel, with an uplifting Weissenberg number. Physically, large relaxation time and fluidic resistance grow faster within converging channels, producing more heat loss and consequently entropy upsurge. While in a narrowing channel, heat loss was subsequently small and hence entropy diminished. 

### 4.3. Consequences of Indexed Power

**Figure 4:**
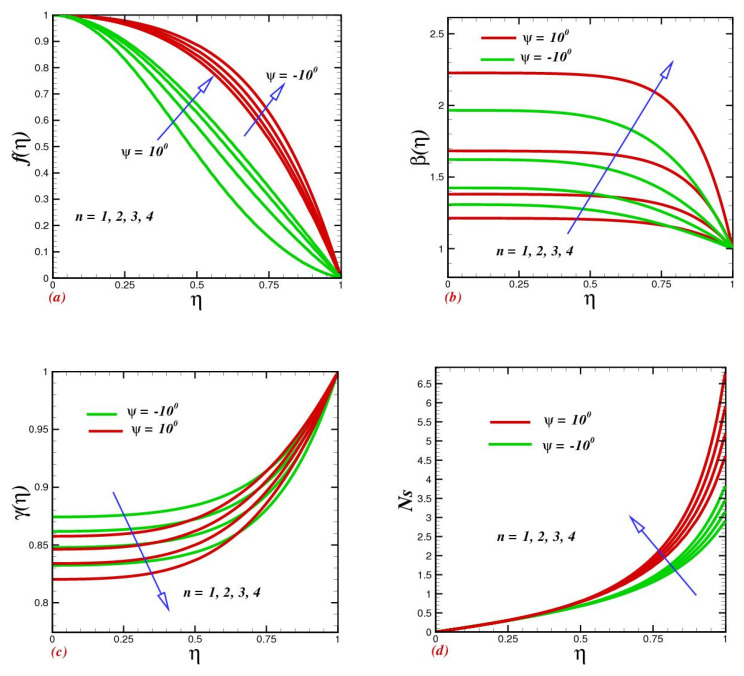
Impact of power-indexed parameter n on (**a**) velocity f(η), (**b**) temperature β(η), (**c**) concentration γ(η), and (**d**) entropy production Ns.

A variety of power-law index parameter values are used in Figure 4 to exhibit the evolution of fluid flow and temperature, concentration, and entropy production rates in distinct fluid channels. Improved power index values cause both the flow and temperature to rise, as seen in these Figure 4a,b. This emerges because the fluid undergoes a shear-thinning to shear thickening transition for higher values of n. Figure 4c conveys the nanoparticle concentration diminutions as the indexed power was enlarged for n>1. In fact, the shear-thickening fluid had a low concentration as compared with the shear-thickening fluid. In addition, the heat transfer and nanoparticle concentration were contrary. Thus, a concentration drop was obvious for non-Newtonian fluid in diverse channels. The heat loss for shear-thickening fluid was more dominant, as clear from Figure 4d. Physically, by improving values of n, the rheological assets of Carreau fluid offer additional confrontation to the nanoparticles drift; as result, more heat loss within diverse channels became dominant, and consequently the system entropy was uplifted. 

### 4.4. Consequences of Magnetic Parameter

**Figure 5:**
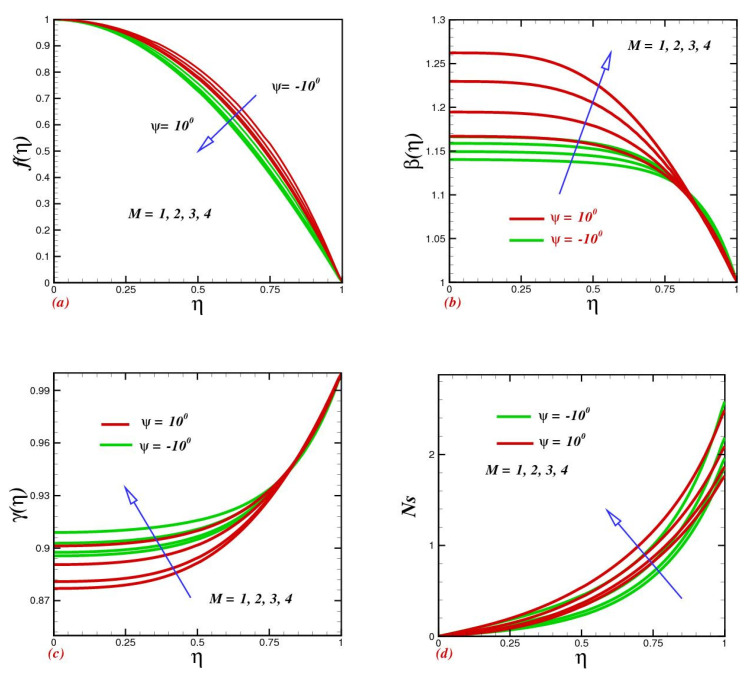
Impact of magnetic parameter M on (**a**) velocity f(η), (**b**) temperature β(η), (**c**) concentration γ(η), and (**d**) entropy production Ns.

Raising the magnetic number M caused a reduction in the channel’s radial velocity, as shown in Figure 5a. It was found that as the magnetic parameter (M) improved, the temperature distribution (Figure 5b) improved while the velocity profile dropped. This is because when M increases, the magnetic field’s Lorentz force also grows and creates more resistance to the flow and nanoparticles. However, the magnetic field raises temperature throughout, causing the thickness of the thermal boundary layer to rise. The reduction of the temperature in the narrowing channel is faster compared with an extended channel. This can be justified by the fact that Lorentz forces suppress the fluid drift and, as a result, the temperature contracts. Concentration uplift for magnetic parameter M strengthening can be witnessed in Figure 5c. The entropy production rate against M is illustrated in Figure 5d. It is seen that magnetic parameters offer a tendency for entropy grooming within the channels. Physically, magnetic field strength suppresses the fluid temperature; as a result, the rheological fluid transmits extra heat to the nanoparticle. Consequently, heat loss ascends within the channel.

### 4.5. Effect of the Eckert Number

**Figure 6:**
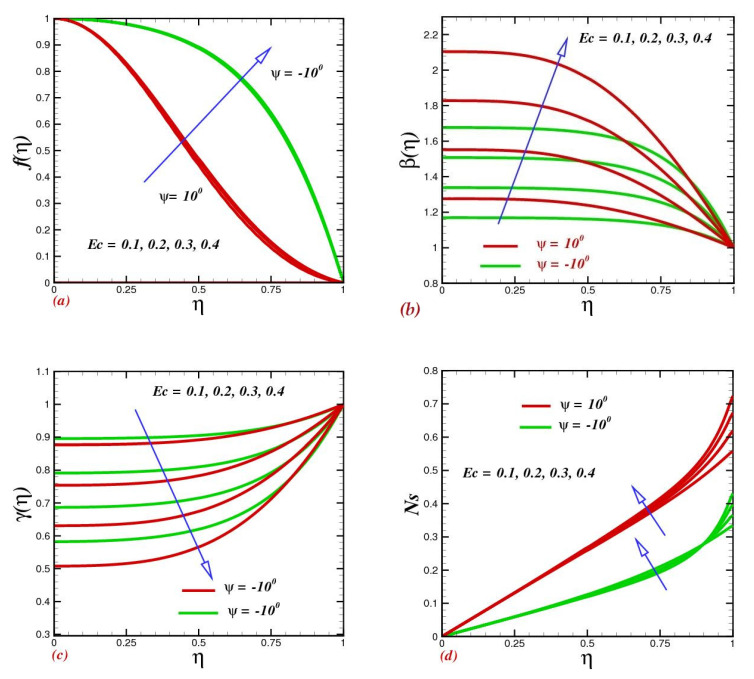
Impact of the Eckert number Ec on (**a**) velocity f(η), (**b**) temperature β(η), (**c**) concentration γ(η), and (**d**) entropy production Ns.

Increasing the Eckert number did not have a significant effect on the fluid velocity, as seen in Figure 6a. In oblique channels, viscous dissipation influenced the velocity a little bit but had a significant influence on temperature, as can be witnessed in Figure 6b. Based on the estimation, the Eckert number endorsed the ratio of the square of maximum velocity and specific heat. Consequently, as the Eckert number rose, the fluid-flow rate along the centerline sped up. For both convergent and divergent channels, Figure 6b reveals that the fluid temperature went up as the viscous heat parameter Ec increased. As a result of the nanofluid’s greater thermal conductivity coefficient, the heat was transported more intensively. Converging–diverging channel nanoparticle concentration trends diminished as the Eckert number Ec increased. In Figure 6d, the Eckert number’s consequence on the system’s irreversibility is examined. Viscous dissipation induced the entropy generation rate Ns to rise massively and consistently along the two hot walls as Ec climbed, as seen in the figure. On the other hand, the positive fluctuation of the Eckert number had a significant influence on the dominating effect of heat-transfer irreversibility at the two heated walls.

### 4.6. Effect of the Brinkman Number on Entropy Generation Rate and the Bejan Number

**Figure 7:**
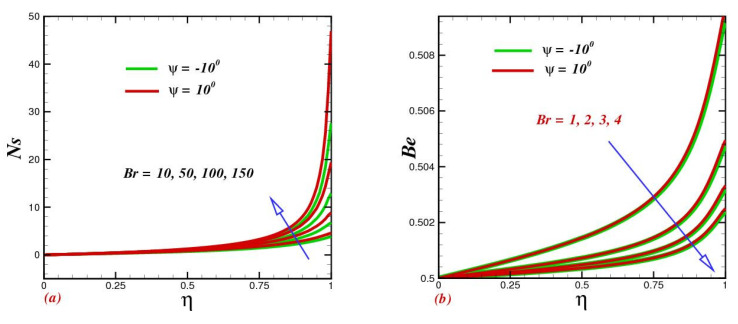
Impact of the Brinkmann number Br on (**a**) entropy production Ns and (**b**) the Bejan profile Be.

The relative contribution of heat generated by viscous dissipation and heat transmitted by molecular conduction is well embodied by  Br. The entropy generation growth within the system of diverse channels against coupled parameter Brinkman number Br is illustrated in Figure 7a. It is related to greater Brinkman numbers in intensifying the fluid friction and heat-transfer rates of the fluid; hence, the entropy generation number significantly upsurges with rising values of Br. Physically, with a higher Brinkman number, the gap in kinetic energy and boundary layer enthalpy increased, owing to which more disturbance developed in the working liquid, and, consequently, the entropy rate rose. The Bejan number Be is determined by the pressure drop along the length of a channel. Physically, it is the connection between the irreversibility of heat transfer and the entire irreversibility produced because of heat transfer. With the enhancement in the Brinkman number, the Bejan profile was significantly lowered. 

### 4.7. Influence of Various Physical Parameters on the Bejan Number

**Figure 8:**
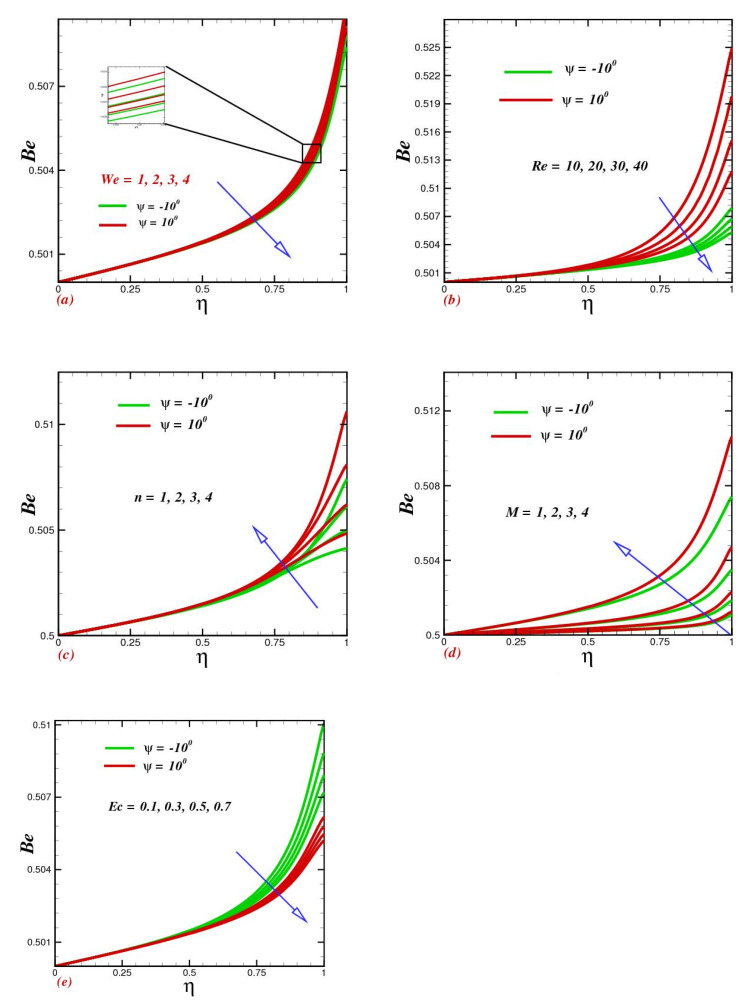
(**a**) Impacts of the Weissenberg number We, (**b**) Reynolds number Re, (**c**) power-indexed parameter n, (**d**) magnetic parameter M, and (**e**) Eckert number Ec on the Bejan profile Be.

The impact of individual thermophysical characteristics on the Bejan number is depicted in the various figures. We found that heat-transfer irreversibility dominated the flow process inside the channel centerline region, with a Bejan number near 1, whereas fluid friction irreversibility had a limited impact on the channel walls. The action of the Weissenberg number on the Bejan profile is illustrated in Figure 8a. Bejan’s curves with improving We seemed to drop. As the the Reynolds number rose, the Bejan number dropped at the converging channel regime to the dominating influence of fluid friction irreversibility and began to rise at the higher-wall region due to the rising effect of heat-transfer irreversibility, as shown in Figure 8b. For diverging channels, the entropy production rates went up at the two walls as Re increased, which is consistent with the findings of flow reversal in that section. The assessment of this graph reveals that increasing parameter n had a substantial impact on improving the Bejan number. Figure 8d highlights the impact of magnetic parameter M on the entropy generation profile. As the magnetic number grew, the liquid temperature went up, enhancing entropy formation. As the fluid temperature went up, the Bejan number near the channel walls went up as well. Furthermore, the major effect of heat transmission irreversibility at the two heated walls illustrated in Figure 8e was influenced by the positive fluctuation of the Eckert number.

### 4.8. Influence of Physical Parameters on Skin-Drag Force and Heat-Transfer Rate

**Figure 9:**
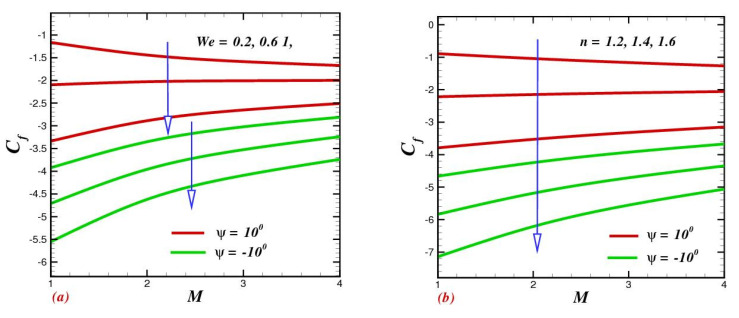
Impact of (**a**) the Weissenberg number We and (**b**) the power-indexed parameter n on skin friction $C_{f}$.

**Figure 10:**
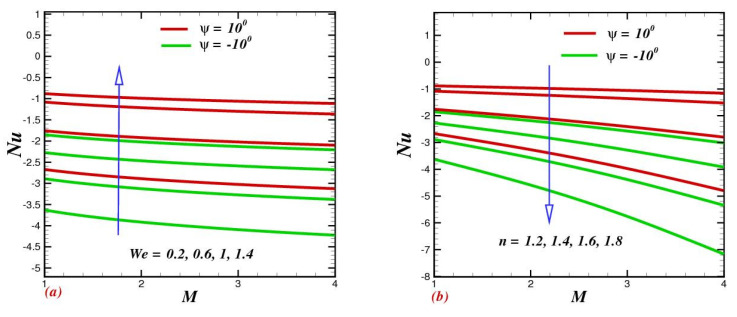
Impact of (**a**) the Weissenberg number We and (**b**) the power-indexed parameter n on the Nusselt number Nu.

The consequences of We and n  on the skin friction coefficient and local Nusselt number are illustrated in Figure 9a,b and Figure 10a,b. Figure 9a exhibits the action of Weissenberg number We on skin friction. It explains that skin friction improved as a function of the applied magnetic field, while a contrary trend was noticed for We. Furthermore, as revealed in Figure 9b, skin friction diminished as the power-law index improved. The action of We against heat-transfer rate is depicted in Figure 10a. The heat-transfer rate was found to be an increasing function of We in oblique channels. Physically, the large amount of relaxation time contributed a significant amount of heat transfer among the nanoparticles in the base fluid; consequently, the Nusselt number improved. The influence of n, when depicted in the view of magnetic field strength, is portrayed in Figure 10b. It was found that the heat-transfer rate dramatically declined with growing n.

Table 1, Table 2 and Table 3 display agreement between our proposed model and the conventional Jaffrey Hamel flow model after implementing certain limitations. We provide a comparison for velocity f(η) in Table 1, the skin force $f_{\eta }\left(1\right)$ in Table 2, and Nusselt number $-\frac{1}{\alpha }\beta_{\eta }\left(1\right)$ in Table 3 for diverse values of angle α; for the channel width, existing data are provided in the literature. An admirable agreement between our numerical approach and existing literature data can be seen, which confirms the accuracy level of our proposed method.

## 5. Conclusions

An entropy production assessment for non-Newtonian hydromagnetic Carreau fluid in the manifestation of viscous dissipations was carried out. The entropy generation rate that arose within the system was calculated using velocity, temperature, concentration, and magnetic field strength. In a converging channel, the flow’s pattern for changing the physical parameters is opposed to that of a divergent channel. The analysis revealed that for the thermally fully developed flow, viscous dissipation had a considerable influence on entropy distribution for higher values of Br (Br > 1), whereas this influence was insignificant for Be. Skin and Nusselt were decreasing functions of power index n. The channel’s walls served as a substantial source of entropy and irreversibility and heat transference. The irreversibility of fluid friction drove entropy production in the channel centerline portion. The flow and heat transmission were controlled by an aligned magnetic field direction. The performance of the Eckert number’s entropy production had a growing influence at the channel’s centerline, and the diverging channel’s rate of heat transfer exhibited the same tendency. The irreversibility of heat and mass transfer dominated the channel’s centerline portion. It is noteworthy to ensure that the two walls caused the system’s overall entropy generation to expand, which had a substantial impact on the heat-transfer rate and velocity profiles. Both divergent and convergent channels experienced increased entropy production, velocity, and heat flux because of channel opening expansion.

## Figures and Tables

**Table 1:** Comparison of numerical values of *f*(*η*) against multiples values of an opening angle *ψ* = 3^0, when *Re* = 4, *We* = 0, Γ = 0 or *n* = 1, *M* = 0.

η	Al-Saif and Jasim [34]	Ghagha et al. [35]	Present Study
0.0	1	1	1
0.1	0.98901	0.98953	0.98953
0.3	0.95626	0.95819	0.95991
0.4	0.90917	0.90619	0.90998
0.5	0.84124	0.83386	0.84001
0.6	0.75123	0.741635	0.74887
0.7	0.64012	0.630019	0.63981
0.8	0.51324	0.499554	0.51018
0.9	0.36129	0.350769	0.35918
1.0	0.19913	0.184134	0.19023
0.3	0	0	0

**Table 2:** Comparison of numerical values of skin friction $f_{\eta }\left(1\right)$ against multiples values of the parameters and an opening angle ψ,  when Re=50, We=1.0, Γ=0 or n=1.

α	$f_{\eta }\left(1\right)$		
	Alam et al. [21]	Rehman et al. [36]	Present Study
$-5^{0}$	−5.13092	−5.13092	−5.13094
	−4.65216	−4.65215	−4.65216
	−2.83395	−2.83391	−2.83393
	0	0	0
$5^{0}$	3.66971	3.66971	3.66963
	−3.50810	−3.50810	−3.50831
	−1.10933	−1.10932	−1.10941
	0	0	0

**Table 3:** Comparison of numerical values of −1/α *β*_*η* (1) against multiples values of an opening angle *ψ*, when *Re* = 50, *Pr* = 3.0, *Nb* = 0.4, *Nt* = 0.2, Γ = 0, or *n* = 1, *M* = 0.

α	Alam et al. [21]	Hayat et al. [33]	Present Study
	0.03157	0.03157	0.03156
$-5^{0}$	0.03734	0.03732	0.03735
	0.04214	0.04215	0.04217
	0.04214	0.04215	0.04213
	0.05052	0.05024	0.05053
$5^{0}$	0.034751	0.03477	0.03474
	0.039993	0.03998	0.03999
	0.046401	0.04640	0.04541

## Data Availability

The author confirms that the data supporting the findings of this study are available within the manuscript and its supplementary files.
